# Altered lipid accumulation in *Nannochloropsis salina* CCAP849/3 following EMS and UV induced mutagenesis

**DOI:** 10.1016/j.btre.2015.05.007

**Published:** 2015-06-05

**Authors:** T.A. Beacham, V. Mora Macia, P. Rooks, D.A. White, S.T. Ali

**Affiliations:** a Plymouth Marine Laboratory, Prospect Place, The Hoe, Plymouth, Devon PL1 3DH, UK; b Plymouth University, Drake Circus, Plymouth, PL4 8AA, UK

**Keywords:** *Nannochloropsis*, Microalgae, Lipid, Random mutation, UV, Biofuels

## Abstract

Microalgae have potential as a chemical feed stock in a range of industrial applications. *Nannochloropsis salina* was subject to EMS mutagenesis and the highest lipid containing cells selected using fluorescence-activated cell sorting. Assessment of growth, lipid content and fatty acid composition identified mutant strains displaying a range of altered traits including changes in the PUFA content and a total FAME increase of up to 156% that of the wild type strain. Combined with a reduction in growth this demonstrated a productivity increase of up to 76%. Following UV mutagenesis, lipid accumulation of the mutant cultures was elevated to more than 3 fold that of the wild type strain, however reduced growth rates resulted in a reduction in overall productivity. Changes observed are indicative of alterations to the regulation of the omega 6 Kennedy pathway. The importance of these variations in physiology for industrial applications such as biofuel production is discussed.

## Highlights

• EMS and UV mutagenesis of *Nannochloropsis salina* combined with FACS for mutant enrichment.
• Productivity of EMS mutants increased by 76% and showed range of FA profile changes.
• Dual EMS and UV mutants accumulated 3 fold more lipid than the wild type.
• Elevation in lipid content comes with a cost to growth rate impacting productivity.
• Mutants suitable for divergent industries generated (biofuel, high value PUFA production).

## 1 Introduction

Despite a slowdown in growth, global energy demand rose 2.3% in 2013 with 32.9% of all consumption derived from oil [5]. Finding alternative sources of liquid transportation fuels to replace the diminishing sources of petroleum oil is crucial to economic stability and development as well as being vital to reduce CO_2_ emissions [23]. In 2012 this translated to a production of biofuels equivalent to 60.22 million tonnes of petroleum oil, representing 1.45% of global oil consumption [5]. The need to find alternative sources of renewable bioenergy and chemical feedstock has in recent years spurred intense interest in the viability of biologically derived oils from sources such as microalgae [22]. Many eukaryotic microalgae have a natural potential to accumulate industrially relevant quantities of triacylglycerols (TAG), neutral lipids that can be converted to fatty acid methyl esters (FAMEs) which are the main component of biodiesel [15]. These photosynthetic microorganisms have low input requirements, higher predicted energy yields per area than terrestrial crops, and the use of marine species would significantly reduce the fresh water impact [6], [9].

In addition to biofuel production, microalgae show promise in production of oils for the aquaculture and healthcare industries since many strains are able to produce high value lipid components such as arachidonic acid (ARA) and eicosapentaenoic acid (EPA). This diversification could help mitigate the large and measurable impact of fish oil production on falling fish populations which is giving rise to both environmental concerns and unsustainable products [12].

Although many microalgae can naturally accumulate TAG in large quantities during the stationary phase of growth [8], [30], actual productivity levels need to be increased in a reproducible manner to make industrial applications for low value products like biofuel production commercially viable [24]. In addition, the suitability of micro algal biomass as biofuel feedstock is closely related to its fatty acid profile with regard to length and degree of saturation which must comply with target ranges defined by the European norm. For many algal species the quantity of high value components such as omega 3 and 6 fatty acids are often at levels too high for fuel specifications but too low for commercial production [28].

Bioengineering offers the possibility of strain development via the introduction of foreign genes to increase lipid production or alter the profile of fatty acids accumulated, pushing towards increasing the poly unsaturated fatty acid (PUFA) content or conversely modifying to produce elevated levels of the saturated medium chain fatty acids (C8–C14) which have properties that mimic current diesel fuels [4]. In practice the number of microalgae species successfully transformed is very low [20], [35] and routine methods for nuclear and organellar transformation severely limited [35]. Furthermore, the availability of multiple genome sequences for both *Nannochloropsis* [26], [32], [36] and *Emiliania huxleyi* [27] has revealed that many microalgae may be subject to extensive genomic variation reflecting their native environment. Consequently, the biochemical and physiological variation amongst strains of the same genus may vary significantly despite near-identical 18S rRNA genes as demonstrated for *Nannochloropsis* [36]. This can complicate targeted modification because considerable variation exists between strains and no two strains will be directly comparable, nor necessarily respond to standard molecular techniques (e.g. antibiotic selection) in the same way. Additionally with targeted modification also comes the need to use selective agents that may be detrimental to the environment and the lengthy and costly legislative requirements involved in GM control, containment and product labelling. Random mutagenesis techniques in contrast are subject to no such controls and over the past 75 years random mutagenesis has been used to create and release more than 2500 plant varieties in 175 plant species, both crop and decorative [29].

In the present study, chemical and physical mutagenesis techniques were used to generate random mutants by exposure to ethyl methane sulfonate (EMS) and UV radiation, respectively. Strain selection via florescence activated cell sorting (FACS) was then used to isolate new strains with enhanced lipid content. Analysis was undertaken to compare two key characteristics deemed to be most desirable: biomass productivity (fast doubling times coupled with growth to high cell densities) and the ability to accumulate a high percentage of biomass as lipid. Considerable variations in productivity were observed across isolated mutant populations of *Nannochloropsis salina* CCAP849/3 strains and the implication of these observations for high and low value oil production is discussed.

## 2 Methods

### 2.1 Strains and culture conditions

*N. salina* algal strain CCAP 849/3 was obtained from the Culture Collection of Algae and Protozoa (Scottish Association for Marine Science, Oban, Scotland, U.K.). Experimental cultures were grown in F/2 medium [13], bubbled with air and maintained under 7400 lux (10.83 W m^2^) irradiance on a 16 h:8 h light:dark cycle at 25 °C. Cells at mid log phase were treated with an antibiotic cocktail of ampicillin (sodium salt) 100 μg mL^−1^, kanamycin monosulphate 100 μg mL^−1^, gentamycin sulphate 50 μg mL^−1^ and streptomycin sulphate salt 100 μg mL^−1^ for 48 h to obtain axenic cultures.

### 2.2 Growth rate determination

Culture density was determined via light microscope cell enumeration in a haemocytometer following staining with Lugols iodine solution (2%). Specific growth rates (*K*) were calculated according to the following equation: *K* = ln(N2/N1)/(t2 − t1), where N2 and N1 are the total cells mL^−1^ at time point (t2) and time point (t1) respectively, and where t2 > t1. Exponential growth refers to cultures ∼5–17 days old. Stationary cultures were >20 days old.

### 2.3 Mutagenesis

For EMS mutagenesis, a method modified from Chaturvedi et al. [7] was used: cells were washed and resuspended at 1 × 10^8^ cells mL^−1^ in fresh sterile sea water with glycerol at 0.1% as a carrier. Cells were subject to EMS (Sigma–Aldrich) exposure at a range of concentrations (0.24 mol L^−1^, 0.42 mol L^−1^ and 0.63 mol L^−1^) over 0.5, 1, 2 and 4 h in darkness at room temperature. EMS was inactivated by addition of sodium thiosulphate solution to a final concentration of 5%. Cultures were pelleted at 8000 g and washed twice with sterile sea water and resuspended in F/2 media. For UV mutagenesis, axenic cultures of wild type 849/3 and two EMS mutagenised lines previously selected for elevated lipid content (mutants NBF6-7 and NBF7-10) were re-suspended at 2 × 10^7^ cells mL^−1^ in fresh F/2 medium. Excess culture (6 mL) was added to a 50 mm petri dish, from which 3 mL was then removed to enable surface tension to form a micro layer of culture spread evenly over the base of the plate. Cells were exposed to UV for 5, 10, 30, 60, 120, 180 and 240 s using the UVP CX-2000 cross linker with a short wave UV source (245 nm) fixed at 89 mm from the sample. Samples from each condition as well as a control were serial diluted and plated for single colonies for determination of cell mortality.

### 2.4 Fluorescence activated cell sorting

Two weeks after EMS treatment and one month after UV treatment, cells were stained with the neutral lipid stain Bodipy 505/515 (Invitrogen) using 0.1% glycerol as a carrier and subject to cell sorting on a Beckman Dickinson FACSAria MkII. Cells were gated on histogram using a blue laser (488 nm) and 530/30 emission filter for the top 5% highest florescence at a rate of 15–30 events per second. EMS cultures were subjected to 3 rounds of sorting, UV cultures to a single round. For EMS mutants only, a final round of sorting was used to initiate monocultures: individual cells were sorted into 96-well plates primed with F/2 media under the same conditions as before but gating limited to the top 1% highest florescence.

### 2.5 Lipid analyses

Fatty acid concentrations and profiles in microalga cells were determined post conversion to fatty acids methyl esters (FAMEs) and analysed by GC–MS (Agilent 7890A GC and 5975C inert MSD, Agilent Technologies Ltd., Edinburgh, UK). Samples were centrifuged (10,000 g), washed in distilled water and resulting pellets lyophilised. Nonadecanoic acid (C19:0) was added as an internal standard and cellular fatty acids were converted directly to FAMEs by adding 1 mL of transesterification mix (consisting a volume to volume ratio of 95% methanolic HCl (3 mol L^−1^) to 5% 2,2-dimethoxypropane) followed by incubation at 90 °C for 1 h. After cooling, FAMEs were recovered by addition of 1 mL NaCl (1%) solution and *n*-hexane (1 mL) followed by vortexing. The upper hexane layer was injected directly onto the GC–MS system and FAMEs were separated on a fused silica capillary column (30 m × 0.2 mm × 0.25 μm; Omegawax™ 250, Supelco, Sigma–Aldrich, Gillingham, Dorset, UK) using an oven temperature initiation of 75 °C for 5 min followed by a gradient increase of 4 °C min^−1^ to 240 °C followed by a hold time of 15 min. Helium was used as a carrier gas (0.4 mL min^−1^) and the injector and detector inlet temperatures were maintained at 280 °C and 230 °C, respectively. FAMEs were identified using retention times and qualifier ion response and quantified using respective target ion responses. All parameters were derived from calibration curves generated from a FAME standard mix (Supelco, Sigma–Aldrich, Gillingham, Dorset, UK).

### 2.6 Statistical analysis

Data was assessed for normality and then subject to ANOVA and 2-sample *T* testing. *P* values of less than 0.05 were considered to be significant.

## 3 Results

Previous work [3] indicated that *N. salina* has a good combination of high oil accumulation and cell proliferation making it an ideal candidate for industrial scale oil production. Using random mutagenesis combined with FACS sorting we aimed to isolate strains improved for production of high and low value oil products. Total FAME accumulation and alterations in fatty acid profile as well as the growth properties of the mutant strains were analysed.

*N. salina* strain 849/3 was subjected to random mutagenesis mediated by EMS. A range of EMS concentrations and incubation times were used to maximise the potential for mutants with alterations in lipid metabolism being recovered. A sample of cells from each of the 12 experimental samples (3 EMS concentration and 4 exposure times) and non EMS controls were serial diluted and plated to determine the approximate cell mortality. Cultures were recovered from all conditions although use of EMS at levels above 0.24 mol L^−1^ for longer than 30 min, caused loss of viability below 3% for this strain. Cultures recovered from the EMS mutagenesis were allowed to recover for 2–4 weeks in F/2 medium such that sufficient cell material was available for FACS sorting.

**Fig. 1:**
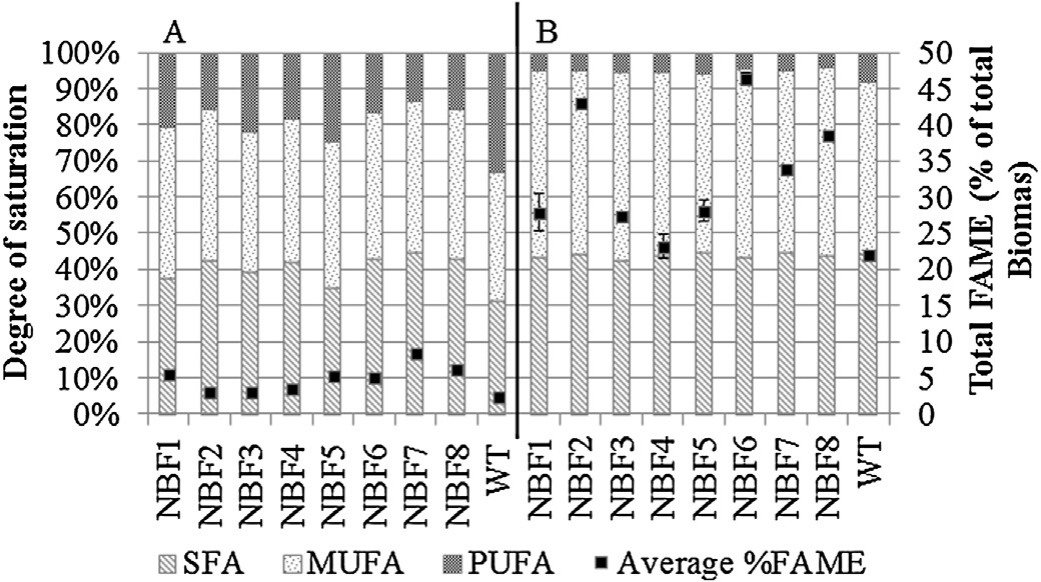
FAME analysis of mixed populations of enriched cultures following EMS mutagenesis and FACS analysis. Panel (a) refers to exponential growth phase; panel (b) refers to stationary growth phase.

Cultures were subjected to 3 rounds of sorting based on fluorescent intensity designed to enrich populations for cells containing the highest level of lipid, predominantly in the form of triacylglycerol. Bodipy 505/515 was used for lipid staining as a better signal is obtained when compared to Nile Red across a range of micro algal species [11]. Enriched cultures were designated NBF1–NBF8 and were subject to FAME analysis during both the active exponential growth phase and at the start of stationary phase (Fig. 1). With the exception of NBF4, total FAME accumulation during the stationary phase was elevated in all mutant populations (*p* = <0.05) compared to the control and varied from 27.9% of total biomass in NBF1 to 46.2% in NBF6. It was also noted that the strains that accumulated FAME at the highest level during the exponential growth phase were not the same as those that had the highest FAME values during stationary phase and may be a consequence of mutations to the control pathway involved in directing lipid accumulation under nitrogen deplete conditions.

Individual fatty acid analysis (Table 1) indicated all mutant populations had reduced levels of PUFAs (*p* = <0.05) compared to the wild type controls during both the exponential and the stationary phases of growth. Elevated levels of saturation are desirable in both traditional FAME biofuel processing and second generation hydrogenation-derived renewable diesel (HDRD) [17]. Of individual fatty acid changes, EPA (C20:5) had the largest reduction within the mutant populations showing a decrease on average of 53% during the exponential phase and 45% during stationary phase when compared to the wild type control. This is similar to a related study [10] on a recently isolated *Nannochloropsis* sp. Since the level of PUFAs drops significantly in both the wild type and mutant as the culture ages, a mutant population with the potential to accumulate high value fatty acids such as EPA would benefit from the elevated total FAME accumulation in early exponential growth as seen for NBF1 and NBF5. This contrasts with populations NBF2 and NBF6 which both show potential for use in biodiesel production where harvesting is likely to be during late exponential or stationary phase to maximise total lipid productivity.

**Table 1:** Fatty acid profile of wild type *N. Salina* and FACs sorted EMS mutated populations. Data expressed as means ± standard deviation (*n* = 4).

		Fatty acids (% total FAME)							
		C14:0	C16:0	C16:1	C18:0	C18:1	C18:2	C20:4	C20:5
Exponential	NBF1	3.1 ± 0.0	31.6 ± 0.2	37.0 ± 0.4	1.3 ± 0.2	4.5 ± 0.0	2.0 ± 0.0	4.2 ± 0.3	13.7 ± 0.3
	NBF2	2.8 ± 0.0	36.5 ± 0.5	36.2 ± 0.3	1.5 ± 0.1	5.2 ± 0.3	1.9 ± 0.1	3.7 ± 0.1	9.3 ± 0.2
	NBF3	3.0 ± 0.0	32.7 ± 0.9	34.7 ± 0.5	1.6 ± 0.1	3.6 ± 0.2	2.0 ± 0.0	5.0 ± 0.1	14.1 ± 0.3
	NBF4	2.9 ± 0.1	36.3 ± 1.0	35.4 ± 0.5	1.4 ± 0.1	5.4 ± 0.4	1.8 ± 0.0	3.8 ± 0.1	10.0 ± 0.2
	NBF5	3.5 ± 0.0	28.9 ± 0.3	36.1 ± 0.4	1.3 ± 0.1	4.0 ± 0.5	2.1 ± 0.0	4.7 ± 0.1	16.7 ± 0.2
	NBF6	2.8 ± 0.0	36.6 ± 0.3	35.8 ± 0.3	1.5 ± 0.0	4.6 ± 0.2	1.7 ± 0.0	4.1 ± 0.1	10.2 ± 0.1
	NBF7	2.4 ± 0.0	38.5 ± 0.5	36.5 ± 0.3	1.7 ± 0.0	5.0 ± 0.3	1.7 ± 0.0	3.2 ± 0.1	7.9 ± 0.1
	NBF8	2.2 ± 0.0	37.1 ± 0.2	36.4 ± 0.3	1.9 ± 0.1	4.5 ± 0.6	2.0 ± 0.0	3.6 ± 0.1	9.3 ± 0.1
	WT	3.9 ± 0.1	24.8 ± 1.0	32.2 ± 1.0	1.1 ± 0.1	3.1 ± 0.6	2.7 ± 0.0	4.8 ± 0.2	24.3 ± 0.3

Stationary	NBF1	1.4 ± 0.0	38.4 ± 0.1	38.3 ± 0.1	1.9 ± 0.0	13.5 ± 0.1	1.8 ± 0.0	0.3 ± 0.2	2.6 ± 0.2
	NBF2	1.4 ± 0.0	38.9 ± 0.3	37.7 ± 0.5	2.0 ± 0.1	13.2 ± 0.2	1.7 ± 0.0	0.4 ± 0.0	2.8 ± 0.0
	NBF3	1.3 ± 0.0	37.5 ± 0.1	37.6 ± 0.3	2.1 ± 0.0	14.4 ± 0.2	2.1 ± 0.0	0.2 ± 0.2	3.1 ± 0.1
	NBF4	1.7 ± 0.0	38.5 ± 1.0	37.2 ± 1.3	1.8 ± 0.1	13.6 ± 0.3	1.6 ± 0.0	0.4 ± 0.3	3.2 ± 0.0
	NBF5	1.9 ± 0.0	38.8 ± 0.1	37.0 ± 0.0	2.0 ± 0.0	12.8 ± 0.0	1.8 ± 0.0	0.4 ± 0.0	3.4 ± 0.0
	NBF6	1.5 ± 0.0	38.3 ± 0.1	37.8 ± 0.2	1.8 ± 0.0	14.6 ± 0.2	1.5 ± 0.0	0.3 ± 0.2	2.8 ± 0.0
	NBF7	1.3 ± 0.0	40.1 ± 0.4	37.5 ± 0.8	1.9 ± 0.0	12.9 ± 0.2	1.7 ± 0.0	0.4 ± 0.2	2.7 ± 0.0
	NBF8	1.3 ± 0.0	39.2 ± 0.5	38.0 ± 0.8	2.1 ± 0.0	13.8 ± 0.1	0.6 ± 1.1	0.2 ± 0.2	3.1 ± 0.1
	WT	2.4 ± 0.1	38.8 ± 0.6	36.5 ± 0.6	1.5 ± 0.1	11.7 ± 0.3	2.0 ± 0.0	0.0 ± 0.0	5.5 ± 0.2

For each of the populations a final round of FACS sorting was used to initiate clonal cultures thus eliminating the observed change in FAME content over time which may arise due to individual variations within a mixed population. Sixty clonal cultures were recovered and subjected to preliminary FAME analysis during exponential phase (data not shown) which enabled identification of mutants with improved oil accumulation during early stage growth and favourable FA profiles. Five diverse clones, NBF7-10, NBF8-3, NBF2-5, NBF6-7, and NBF7-3 were chosen for more detailed analysis, based on a combination of the total FAME content and different degrees of fatty acid poly-unsaturation.

The lipid content of *N. salina* CCAP 849/3 steadily increases during the active growth phase. Since continuous cultivation and harvesting is desirable on an industrial scale, ideally the harvesting will need to take place late enough to maximise recovery of commercially relevant quantities of oil but without compromising growth. Lipid analysis to measure the FAME content and composition was therefore performed during late exponential growth.

**Fig. 2:**
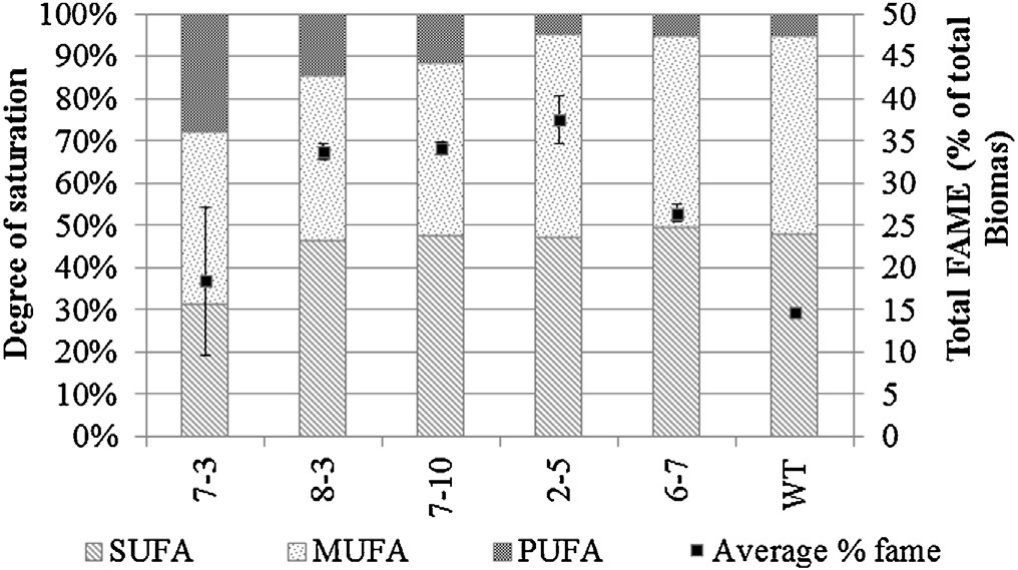
FAME analysis of EMS mutants during late exponential grow phase. Total FAME expressed as means ± SEM (*n* = 4).

Total FAME content (Fig. 2) showed considerable diversity from 18.3% of total biomass in strain NBF7-3 to 37.5% of dry biomass in strain NBF2-5; more than double that of the wild type strain under these growth conditions. Comparison of data in Fig. 1, Fig. 2 highlights the value of clonal selection with big differences observed for example between the mixed population NBF6 and the clonal isolate NBF6-7. Variation of overall fatty acid saturation between the strains was also relatively high. Two strains (NBF6-7 and NBF2-5) maintained a comparable level of saturation to the control whilst the other three showed increasing levels of poly-unsaturation between 2 and 5.5 times that of the wild type strain.

**Fig. 3:**
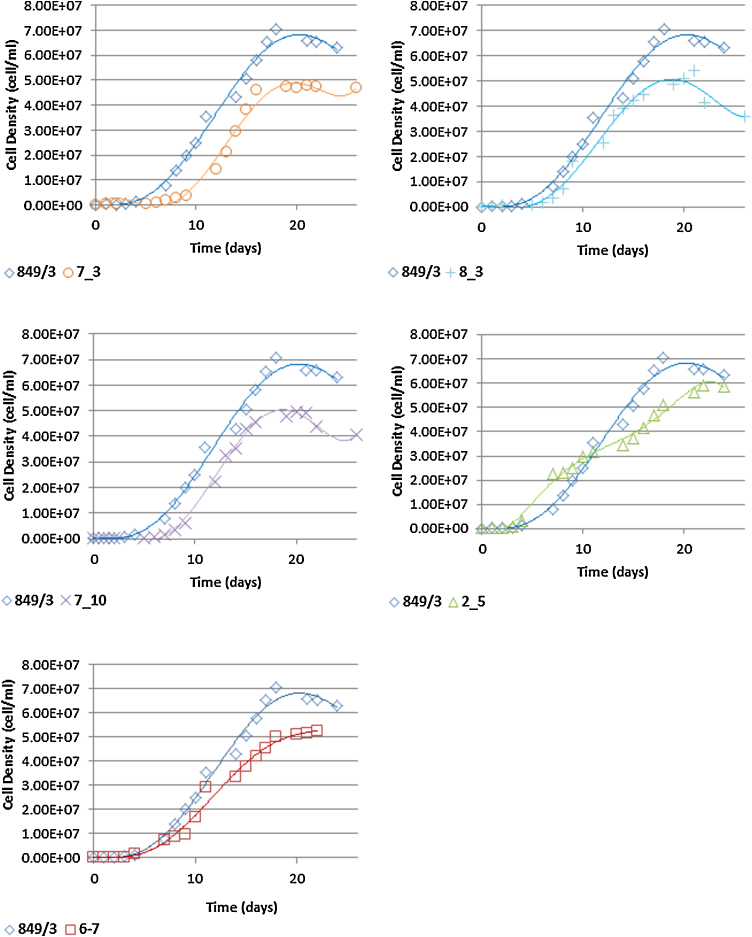
Growth characteristics of EMS mutants over a 26 day period. Specific exponential growth rates are: WT = 1.27, NBF7-3 = 0.77, NBF8-3 = 1.25, NBF7-10 = 1.04, NBF2-5 = 0.11*,* NBF6-7 = 0.74.

Strains NBF2-5 and NBF6-7 showed no significant alteration from the control within the individual fatty acid profiles (Table 2) with palmitic acid (C16:0) and sapienic acid (C16:1) comprising approximately 70% of the total FAME. Elevation in the amount of lipid that the algae accumulates often comes with a cost to growth [31]. In the case of the 5 clonal strains assessed here, NBF6-7, NBF2-5, NBF7-10 and NBF7-3 showed a significant reduction in growth rate, and all had reduced maximum cell density as shown in Fig. 3. This loss in biomass and increase in time to harvest needs to be considered as it could negate any improvement in lipid content. Strain NBF2-5 achieved a maximum cell density of 5.9 × 10^7^ cells mL^−1^ over 22 days. With FAME content of 8.3 pg cell^−1^ (37.48%) this would equate to a productivity of 22.3 μg mL^−1^ day^−1^. Despite the reduction in growth rate this still compares favourably to the wild type which achieved a maximum cell density of 7.0 × 10^7^ cells mL^−1^ over 18 days. With a FAME content of 3.2 pg cell^−1^ (14.61%) this equates to a productivity of 12.62 μg mL^−1^ day^−1^; 46% less than that attained by the mutant strain.

**Table 2:** Fatty acid profile (major fatty acids) of wild type *N. Salina* and FACs sorted clonal EMS mutated strains during late exponential growth phase. Data expressed as means ± standard deviation (*n* = 4).

	Fatty acids (% total FAME)							
	C16:0	C16:1	C18:0	C18:1	C18:2	C18:3	C20:4	C20:5
NBF7-3	14.4 ± 2.4	10.0 ± 17	3.9 ± 2.2	29.6 ± 16	3.1 ± 1.7	1.6 ± 0.9	12.1 ± 6.8	9.5 ± 5.3
NBF7-10	41.5 ± 0.4	30.3 ± 0.4	1.6 ± 0.0	9.8 ± 0.1	1.3 ± 0.0	0.6 ± 0.0	4.9 ± 0.0	4.2 ± 0.1
NBF8-3	40.3 ± 0.7	29.7 ± 1.0	2.0 ± 0.0	8.6 ± 1.6	1.8 ± 0.0	0.7 ± 0.0	4.8 ± 1.7	6.5 ± 0.2
NBF6-7	43.4 ± 1.6	33.0 ± 2.1	1.6 ± 0.0	11.8 ± 0.9	1.6 ± 0.4	0.6 ± 0.1	2.6 ± 0.0	0.0 ± 0.0
NBF2-5	42.1 ± 0.7	34.9 ± 0.3	1.5 ± 0.1	12.3 ± 0.7	1.3 ± 0.3	0.5 ± 0.0	2.5 ± 0.2	0.0 ± 0.0
WT	42.5 ± 0.7	34.5 ± 0.5	1.5 ± 0.1	11.9 ± 0.4	1.6 ± 0.3	0.6 ± 01	2.5 ± 0.1	0.1 ± 0.0

Strain NBF7-3 was very rich in very long chain PUFAs, particularly the high value omega 6 EPA (C20:5) and ARA (C20:4) fatty acids which made up a total 21.65% of the total FAME content compared with just 2.6% in the WT strain (Table 2). The lack of significant improved oil accumulation combined with the highly unsaturated nature of the FAME components make this strain particularly unsuited to biofuel production. Conversely, the change in saturation does however make NBF7-3 potentially good for high value PUFA production.

**Fig. 4:**
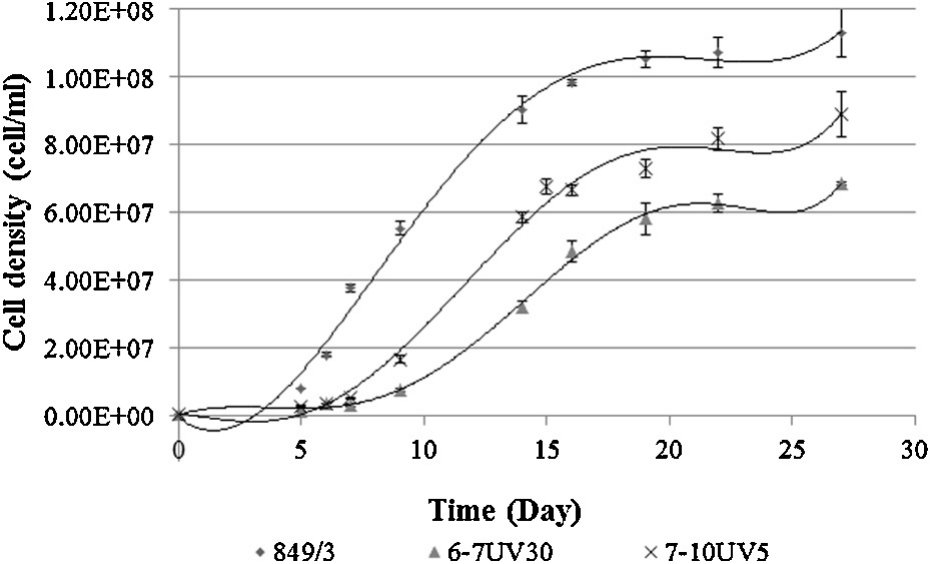
Comparison of growth between wild type and dual (EMS + UV) mutagenized strains of *Nannochloropsis*. Data fitted with 6 order polynomial trend line and error bars SEM (*n* = 4).

Two mutant strains chosen for their disparate degree of saturation compared to the wild type control (NBF6-7—elevated level of saturation; NBF7-10—elevated level of poly unsaturation) were subjected to shortwave UV radiation to induce a further round of mutation. Cell mortality was very high for both cultures following UV exposure and an accurate viability calculation was not possible. No cells were recovered after exposure to UV for longer than 30 s. Phenotypic assessment (initial growth and cell morphology) of the recovered populations was used to identify two populations for further assessment. Phenotypic analysis assessed the changes in growth and maximum cell density achieved over a 27 day period during which all cultures entered stationary growth phase (Fig. 4).

Both dual mutated populations had significantly reduced growth rates compared to the control wild type (CCAP 849/3) strain (*p* = <0.05) with NBF6-7UV30 and NBF7-10UV5 exhibiting a 95% and 56% reduction in growth, respectively. In addition, an increased lag time combined with a reduction in the time spent in exponential growth negatively impacted on the maximum cell densities achieved in the mutant strains (Table 3) with NBF6-7UV30 being particularly poor even compared to NBF7-10UV5 (*p* = <0.05). The growth alacrity of both mutant populations was also reduced when compared to the EMS parent strains from which they were generated. This could be due in part to an increasing number of negative mutations affecting the cells as well as a slowing of reproduction in favour of oil accumulation during the cell filling stage.

**Table 3:** Productivity analysis of EMS mutagenized and dual mutagenized (EMS + UV radiation) strains. Total FAME (% of biomass) and Fatty acid profile relates to sampling at mid exponential phase. Data expressed as means ± standard deviation (*n* = 4).

	Exponential growth rate relative to WT (%)	Max cell density (cell/mL)	Total FAME (%)	Max. FAME productivity (μg mL^−1^ day^−1^)	Fatty acid profile		
					SUFA	MUFA	PUFA
WT	100.0	1.1.E + 08	17.5 ± 0.3	44.5	39.0 ± 0.2	35.0 ± 0.1	26.0 ± 0.1
NBF6-7	57.8	5.3.E + 07	26.4 ± 1.1	33.3	49.6 ± 1.8	45.2 ± 1.2	5.1 ± 0.6
NBF6-7UV30	5.1	6.9.E + 07	57.3 ± 5.1	35.2	40.0 ± 4.3	44.0 ± 5.9	16.0 ± 1.6
NBF7-10	81.8	4.9.E + 07	34.1 ± 0.8	49.0	47.8 ± 0.3	40.7 ± 0.0	11.5 ± 0.3
NBF7-10UV5	44.2	8.9.E + 07	78.7 ± 6.2	37.1	47.0 ± 4.2	34.0 ± 5.8	19.0 ± 1.6

Lipid analysis during the mid-exponential phase of growth showed a significant elevation in the total FAME accumulation (Table 3) in the dual mutants compared to the control, however this gain only partially offset the reduced biomass accumulation and resulted in an overall 16.6% and 20.9% reduction in maximum productivity in NBF7-10UV5 and NBF6-7UV30, respectively as compared to the control strain (CCAP 849/3). Analysis of fatty acid composition and degree of saturation showed a reduction in PUFAs in both NBF7-10UV5 and NBF6-7UV30 compared to the control. In NBF7-10UV5 this shift was associated with a significant (*p* = <0.05) increase in saturated fatty acids, particularly palmitic acid. In contrast NBF6-7UV30 had no change in the level of saturated fatty acid (SFA) but had a significant increase in the level of monounsaturated fatty acids (*p* = <0.05), particularly oleic acid.

Given that the properties of the various individual fatty esters determine the overall properties of biodiesel [18], the fatty acid profile, depending on seasonal requirements, make oil from both these dual mutants suitable as biodiesel feed stock.

## 4 Discussion

### 4.1 EMS mutants

Following EMS treatment, an examination of the initial mixed mutant population strains would suggest that in all 8 populations (NBF1–NBF8) the dominant effect is an accumulation of C16:0 and concomitant reduction in the synthesis of C20:5 during active growth. No significant change in the levels of other fatty acids of intermediate chain length/structure occurred suggesting the dominant mutation in these populations specifically affects the biosynthesis of C20:5 FA during active growth (Table 1). It is unclear what the biochemical basis for this observation is; it is interesting to note that Hamilton et al. allude to the existence of alternative biosynthetic enzymes in microalgae [14] and this is further highlighted by the recent sequencing of several *Nannochloropsis* genomes which reveal the existence of multiple candidate genes for fatty acid biosynthesis indicative of a high level of complexity for these pathways.

Further purification of individual clones from these mixed populations (Table 2) has resulted in the isolation of two distinct groups: strains NBF7-3, NBF7-10, and NBF8-3 have elevated C20:4 and C20:5 which is contradictory of the trend seen in the mixed populations; and NBF6-7 and NBF2-5 which have a composition very similar to the wild-type strain with little PUFA present. All strains (except NBF7-3) show a significant increase in their total lipid levels when compared to the control (Fig. 2, black squares) suggesting that lipid biosynthesis is generally elevated in these cells.

**Fig. 5:**
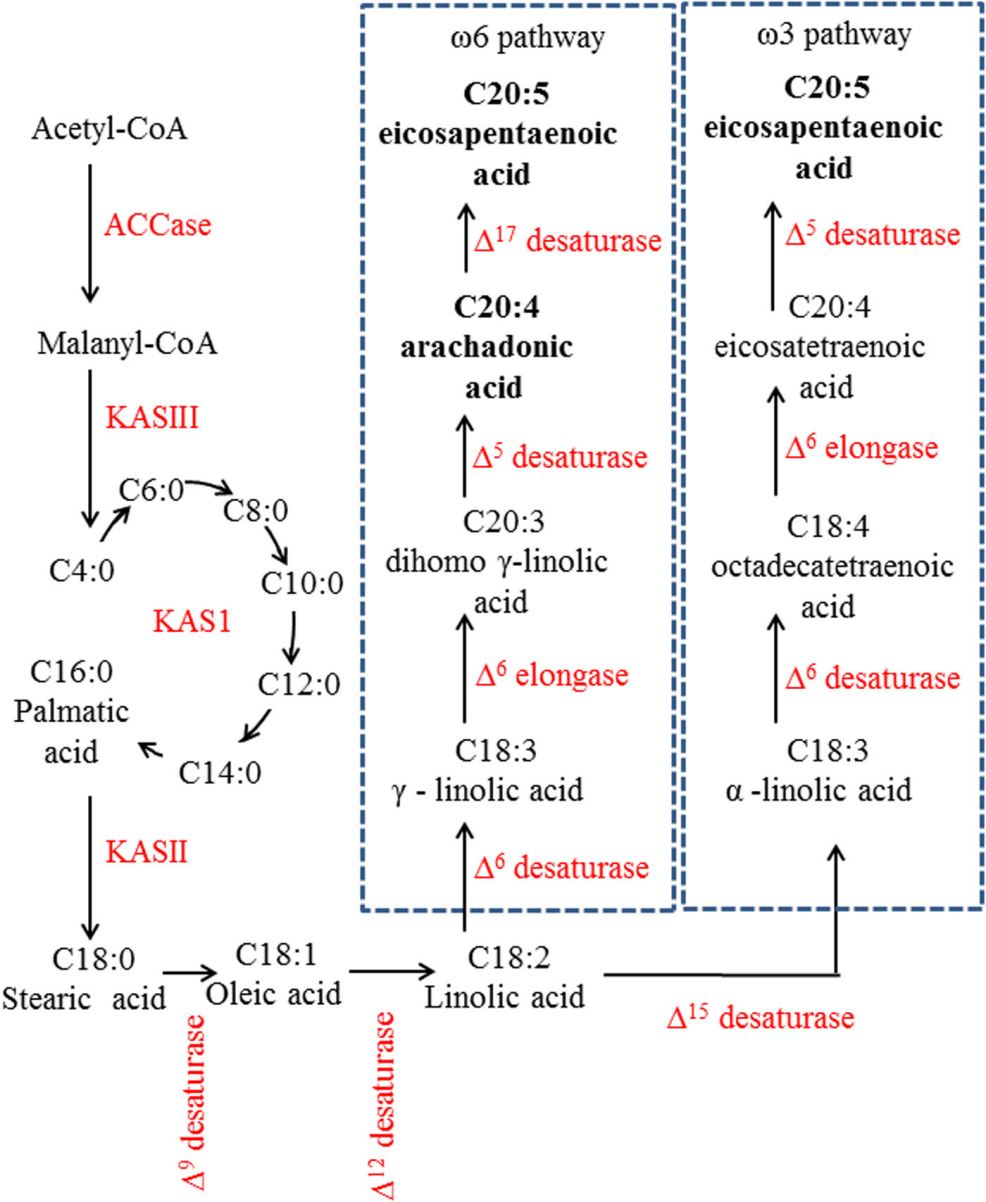
Fatty acid biosynthesis acid in eustigmatophytes—modified from Guschina and Harwood (2006) and Napier (2007).

ARA and EPA accumulation varies during the life cycle of wild type *Nannochloropsis* from levels in excess of 15% of the total FA pool during exponential growth to almost undetectable levels during stationary phase. This indicates the biosynthesis pathways involved have a natural flexibility in the capacity to form these very long chain fatty acids (VLCFA). It is unlikely that a simple up-regulation or enhancement of one or more of the desaturase and elongases in the omega 6 pathway (Fig. 5) would be sufficient to induce the FA profile change observed in mutant strains such as NBF7-3. We speculate that a deregulation of the fatty acid synthase KASII is a likely candidate for the substantial increase in conversion of palmitic acid to stearic acid observed, thus altering the available substrate pool for downstream biosynthesis. Whilst this is likely to produce some substrate controlled flux toward VLCFA production it does not fully explain the metabolic changes of this particular mutant, since elevating the amount of substrate at the start of the biochemical pathway will not necessarily produce an increase in downstream FA production. Table 2 shows, for example, that wild type *N. salina* in stationary phase has an abundance of oleic acid compared to exponential phase yet this is not translated in to VLCFA, thus implicating a highly regulated conversion of oleic acid to linoleic acid via Δ12 desaturase as the most obvious metabolic control node. This suggests that NBF7-3 has a form of Δ12 desaturase deregulation, and potentially one or more changes within the downstream omega 6 biosynthetic pathway. These alterations are then preventing the down regulation of this pathway in favour of the short chain storage lipids that are normally produced in late exponential growth as observed in WT strains.

For strains NBF7-10 and NBF8-3 we observed a smaller pull toward formation of ARA and EPA than in NBF7-3 and since there is a reduction in the oleic FA pool within these mutants compared to the control, this pull is not due to substrate controlled flux, but rather a similar deregulation in Δ12 desaturation as speculated for NBF7-3. Additionally, with NBF8-3 mutations favouring the production of EPA over ARA we speculate that this could indicate an up-regulation of the Δ17 desaturase of the omega 6 pathway. Since we found only very low levels of alpha linolenic acid and no detectable levels of 18:4 (n3) in any of our mutant strains we do not think that the elevation in EPA is due to enhanced production along the omega 3 pathway.

The first committed enzyme in the fatty acid biosynthetic pathway is acetyl-CoA carboxylase (ACCase), an enzyme that is both highly regulated and a key regulator of flux of carbon into fatty acid synthase [1], [2]. We have observed here that several of our mutant strains show dramatic elevations in total lipid compared to wild type.

Observations by Chaturvedi et al. [7] showed that an increase in acetyl-CoA carboxylase (ACCase) specific activity not only accompanies increasing lipid content but also confers a resistance to the herbicide quizalofop and an elevated thermal tolerance. When subjected to quizalofop or elevated temperature none of our mutant strains demonstrated any increased resistance compared to the controls (data not shown), suggesting that alterations to ACCase activity are unlikely to be responsible for the elevation in lipid accumulation observed here.

Glycerol-3-phosphate acyl transferase (GPAT) catalyses the initial step in triacylglycerol (TAG) and phospholipid biosynthesis and research in both mammalian [21] and plant subjects [34] have shown that elevated GPAT activity results in an increase in TAG accumulation where substrate is not limiting, and that GPAT is a major metabolic control node in the stimulation of flux into TAG production [33], [34]. At the end of the Kennedy pathway it has been suggested that DGAT is the rate limiting step in TAG assembly [25] and it has been shown that overexpression of this enzyme also leads to elevated TAG accumulation [16], [19]. We suggest therefore, that mutations to one or more of the enzymes in the Kennedy pathway (or those involved in its regulation) are likely to be responsible for the increase in total FAME production observed in many of our mutant strains.

### 4.2 Dual mutants

Attempts to further alter the lipid biosynthesis in these EMS mutants using UV irradiation generated mixed results. Cell viability was greatly compromised upon exposure to short wave UV light for even short periods of time. This is surprising given that *Nannochloropsis* is a phototrophic organism that would be naturally resilient to exposure to high levels of natural UV (sunlight) for extended periods in the environment. Two possible explanations could account for this UV sensitivity. CCAP 849/3 has become adapted to low light conditions in culture and no longer responds well to UV exposure or that *Nannochloropsis* naturally occurs in deeper waters in the marine environment (the deep chlorophyll maximum) and has not evolved a strong defence mechanism for UV light due to the protective attenuation effect of the ocean. It is interesting to note that CCAP 849/3 grows more vigorously in low light conditions than in high light conditions (Beacham and Ali, unpublished observation). Only two strains (6-7UV30 and 7-10UV5) were successfully recovered from UV mutagenesis experiments and both showed decreased growth rates compared to the wild-type control but with significantly elevated levels of total lipid and a reduction in PUFA, indicative that changes in lipid metabolism have occurred and making these strains suitable for biofuel production. In addition these 2 mutants demonstrate the common problem associated with altering the natural distribution of cell resources; achieving the desired change (for example increased FAME accumulation) often comes with a negative pay off (in this instance a reduction in the growth rate). As such clones may need to be selected for not by the gross amount of a compound they can produce but by how much the overall productivity has been enhanced.

## 5 Conclusion

As we have observed here the untargeted nature of random mutation can have undesirable effects on growth and cell function and as such the level of mutation must be strictly managed by intensive strain screening and selection. The changes observed could be the result of alterations at the transcriptional level and/or enzymatic level and further analysis is required to understand the molecular mechanisms involved in the modified biochemical pathways observed. A systematic transcriptomic and genomic sequencing analysis of the different mutants generated in this study would yield valuable insights into lipid metabolism in these potentially valuable industrial organisms. This work demonstrates classical mutagenesis techniques can be successfully used to manipulate both the lipid productivity and FA composition, for multiple divergent industrial applications such as biofuel or high value oil production without the need for directed gene modification. These methods also alleviate the need to use selective agents that may be detrimental to the environment and the lengthy and costly legal requirements involved in GM control, containment and product labelling. The techniques described could also easily be adapted for production of other industrially relevant components such as pigments by changing the enrichment selection parameters.
